# Master lineage transcription factors anchor *trans* mega transcriptional complexes at highly accessible enhancer sites to promote long-range chromatin clustering and transcription of distal target genes

**DOI:** 10.1093/nar/gkab1105

**Published:** 2021-11-24

**Authors:** Shannon M White, Michael P Snyder, Chunling Yi

**Affiliations:** Lombardi Comprehensive Cancer Center, Georgetown University Medical Center, Washington, DC, USA; Department of Genetics, Stanford University, Stanford, CA, USA; Department of Genetics, Stanford University, Stanford, CA, USA; Lombardi Comprehensive Cancer Center, Georgetown University Medical Center, Washington, DC, USA

## Abstract

The term ‘super enhancers’ (SE) has been widely used to describe stretches of closely localized enhancers that are occupied collectively by large numbers of transcription factors (TFs) and co-factors, and control the transcription of highly-expressed genes. Through integrated analysis of >600 DNase-seq, ChIP-seq, GRO-seq, STARR-seq, RNA-seq, Hi-C and ChIA-PET data in five human cancer cell lines, we identified a new class of autonomous SEs (aSEs) that are excluded from classic SE calls by the widely used Rank Ordering of Super-Enhancers (ROSE) method. TF footprint analysis revealed that compared to classic SEs and regular enhancers, aSEs are tightly bound by a dense array of master lineage TFs, which serve as anchors to recruit additional TFs and co-factors *in trans*. In addition, aSEs are preferentially enriched for Cohesins, which likely involve in stabilizing long-distance interactions between aSEs and their distal target genes. Finally, we showed that aSEs can be reliably predicted using a single DNase-seq data or combined with Mediator and/or P300 ChIP-seq. Overall, our study demonstrates that aSEs represent a unique class of functionally important enhancer elements that distally regulate the transcription of highly expressed genes.

## INTRODUCTION

Enhancers are short, accessible genomic loci that distally regulate the transcription of target genes that are in some cases located hundreds of kilobases away (1). In a highly-regulated and motif-dependent manner, enhancers are recognized and directly bound by a myriad of TFs, which facilitate the recruitment of additional TFs, co-activators and/or co-suppressors to these sites *in trans*. Through chromatin looping, these enhancer-bound transcriptional complexes can make physical contact with other enhancers and/or promoters, and dynamically regulate the transcriptional initiation and elongation of the target genes (1). Highlighting the physiological importance of enhancer elements, disease-associated genetic variants including well-established cancer-predisposing alleles are frequently enriched within enhancer regions (2,3).

There are estimated to be thousands of active enhancers in a given mammalian cell, many of which function cooperatively to regulate target gene expression (4,5). Earlier chromatin profiling studies described the existence of stretches of closely located enhancers with similar chromatin accessibility, which were termed clusters of regulatory elements (COREs) (6,7). Subsequent studies showed that certain large clusters of enhancers, often spanning tens of thousands of kilobases, collectively drive high-level expression of lineage-specific genes, and classified these groups of enhancers as SE (8–10). Compared to regular enhancers (rEh), SE exhibit disproportionately higher binding signals of the mediator complex subunit MED1, the histone acetyltransferase p300, and the bromodomain and extra-terminal motif (BET) family member BRD4, as well as increased levels of H3K27ac, H3K4me1 and DNase I hypersensitivity (8–11). In addition, SEs have been shown to produce high levels of bi-directional, short-lived enhancer RNA (eRNA) transcripts, which play active roles in maintaining enhancer activities (12–14).

A number of seminal studies have found that SEs drive the expression of lineage-specific master TFs, which in turn preferentially bind to and activate SEs, forming positive feedback loops or so-called core transcriptional networks in a broad range of normal and cancer cells (8,9,15,16). During cancer initiation and progression, the SE landscape has been shown to undergo extensive remodeling (17–19), which is further altered by drug treatments (20–22). Based on the tendency of master oncogenic TFs to bind to and be regulated by SEs, a number of studies have successfully utilized motif enrichment coupled with transcript proximity at SEs to predict candidate master oncogenic TFs (11,19,23–25).

Concerted efforts by the Encyclopedia of DNA Elements (ENCODE) consortium, the Roadmap Epigenetic Project and individual groups have generated chromatin immunoprecipitation followed by sequencing (ChIP-seq) data for hundreds of TFs in multiple widely-used cell lines. Comparative analysis of TF binding profiles within the same cell lines revealed that certain genomic loci, commonly referred to as high occupancy target (HOT) sites, are bound by dozens to hundreds of TFs and co-factors (26–31). While some studies posited this phenomenon as technical artifacts caused by high DNA accessibility or GC-rich sequences (32,33), increasing evidence indicates that the HOT sites are functional genomic loci that drive the transcription of highly expressed, functionally important target genes (26–30). Notably, the majority of HOT sites fall outside the classically defined SE regions (27), suggesting that distinct mechanisms and factors likely drive the *trans*-assembly of mega transcriptional complexes at these stand-alone sites.

Through integrated analysis of >600 publicly available DNase-seq, ChIP-seq, Global nuclear run-on sequencing (GRO-seq) and three-dimensional (3D) chromatin interaction data from five commonly used cancer cell lines, we re-defined *trans*-acting high-occupancy enhancers considering of both the numbers of factors bound *and* the relative signal intensities for each factor. Our systematic analysis revealed that the re-defined high-occupancy enhancers, while physically distinct from the classic SE, exhibit all the expected functional characteristics of SEs. We further showed that even without chromatin-binding data from a large number of TF and co-factors, *trans*-acting high-occupancy enhancers can be reliably identified by ranking the signals from DNase-seq, and/or ChIP-seq of several commonly studied transcriptional co-activators. Based on the results of our analysis, we propose to expand the SE definition to include trans high-occupancy enhancers, and re-divide SE into three subclasses of *cis*, *trans* and dual SE, reflecting their distinct modes of actions.

## MATERIALS AND METHODS

### ChIP-seq data collection and processing

All available TF and histone ChIP-seq processed datasets aligned to the human reference genome (GRCh38) were downloaded from the ENCODE data portal (see Supplementary Table S1 for ENCODE experiment references). To expand the repertoire of ChIP-seq data, Cistrome Data Browser (34) was used to identify high quality public ChIP-seq data for TFs, cofactors, and histone marks that were not included in the ENCODE database. All non-ENCODE datasets (see Supplementary Table S1 for Sequence Read Archive (SRA) references) were processed using the standardized ENCODE-DCC pipeline available on Github (https://github.com/ENCODE-DCC/chip-seq-pipeline2) according to their recommended guidelines. Briefly, raw fastq files were aligned to GRCh38 using the Burrows Wheeler Aligner (BWA, v0.7.17) algorithm (35). Post-alignment filtering was conducting using Samtools (v1.9) (36) to filter alignments using a MAPQ threshold of 30 and picard-tools-2.10.6 (https://broadinstitute.github.io/picard/) to remove duplicates. Peak calling was then carried out using the SPP algorithm, where peak rankings and replicate consistency were determined using the irreproducible discovery rate (IDR) package (37,38). The resulting optimal narrowpeak files or replicated peak files for TF and histone datasets, respectively, were used for future analyses.

### ChIP-seq data enhancer classification

#### Enhancer identification

To identify active enhancers in each cell line, we first subsetted DNase-seq peaks that overlapped with H3K27ac ChIP-seq peaks using findOverlapsOfPeaks from the ChIPpeakAnno R package (39,40). The resulting H3K27ac + DNase peaks were further excluded for promoters by removing any peaks located within +/−2.5 kb from any transcription start site (TSS), calculated using annotatePeakInBatch from the ChIPseeker R package (41). The DNase peaks that passed both steps of filtering were defined as active enhancers and used for all downstream analysis (see Supplementary Table S2 for detailed statistics of peaks retained throughout all the steps of filtering).

#### Identification of super high occupancy enhancers

To assess the binding occupancy and strength of all TF, cofactors, and histone marks at all active enhancer regions, we used ChIPpeakAnno R package (39,40) to overlap all ChIP-seq peaks with the active enhancer peaks resulting in a final matrix displaying the active enhancer regions (rows) and the corresponding signal value of each factor (columns) at that enhancer peak. For all enhancer regions where a factor did not have an overlapping peak the signal value was assigned to zero. Signal values for each factor were normalized using min-max normalization, resulting in a signal value range for all factors between 0 and 1. To calculate the occupancy score (OS) only TF and cofactor ChIP-seq data was considered (histone data was excluded). The OS was calculated using the following formula:

$$\begin{equation*}{OS(E)}=N_Ex \sum_f{Signal_E }\end{equation*}$$

where *N* is the number of factors, *f*, bound at enhancer, *E*. The calculated OS for each enhancer is dependent on the number of factors bound and the signal strength of each bound factor, as indicated by the sum of the factor signal values. By adapting the ROSE R script (https://bitbucket.org/young_computation/rose/src/master/), enhancers were ranked according to their OS values and plotted. The OS score at the curve's inflection point was deemed the cutoff and all enhancers with an OS score greater than the cutoff were classified as high occupancy enhancers.

#### Defining aSE, cSE and dSE

The ROSE algorithm (8,10) was utilized to identify classic SE regions using H3K27ac ChIP-seq data under default settings (stitching distance: 12.5 kb; tss_exclusion_zone_size: 0). Next, H3K27ac + DNase peaks that overlapped with the classically defined SE regions were extracted. Those also meeting the cutoff of super high occupancy enhancers from above were classified as dSE, and the rest were named cSE. Finally, the super high occupancy enhancers did not overlap with the classically defined SE regions were renamed as aSE. Of note, as substantially fewer TF and cofactor ChIP-seq datasets are available for A549 and HCT116, super high occupancy enhancer classifications are less definitive in these two cell lines relative to HepG2 and MCF7.

### DNase-seq footprinting and motif analysis

To identify high-confidence cell line-specific TF footprints, we filtered cell line-specific footprints (with a false discovery rate of 0.01) for footprints present in the ENCODE consensus footprint dataset, a high-confidence dataset identified by analyzing DNase-seq profiles across 243 human cell and tissue types (42). Consensus cell type-specific footprints were then overlapped with enhancer class peaks to calculate the number of TF footprints per enhancer peak, footprint width, motif bitscore, and distance between footprints within the same enhancer peak. To eliminate redundancy within motifs, motifs were classified based on their DNA binding domain (DBD) family. Footprints with multiple matched DBD family motifs were assigned to one DBD family based on the highest motif bitscore value. To determine direct TF binding, all enhancer class peaks were annotated with the DBD families present in the overlapping footprints. For all TF ChIP-seq peaks that overlap enhancer peaks, if the corresponding enhancer peak was annotated with a matching DBD family footprint, the TF was considered to bind directly to that enhancer peak, otherwise the TF was marked as binding indirectly. All bar and scatter plots were generated using the R package ggplots2 (43). To generate the DNase-seq signal profile plots for individual motif footprints, we utilized the Hmm-based IdeNtification of Transcription factor footprints (HINT) software (44).

### GC, CpG, and conservation score analyses

Homer (v4.11) software was used to calculate GC and CpG percentages for enhancer class peaks. Vertebrate phastCon scores for the GRCh38 genome were downloaded from UCSC table browser (45,46). All plots were generated using the R package ggplot2 (43).

### DNase-seq peak width calculation

To calculate the unrestricted peak width for all DNase-seq regions, we re-called DNase-seq peaks to permit peaks of variable widths using the findpeaks -region option from the Homer peak-calling software (47). The newly-called variable-width DNase-seq peaks were overlapped with all enhancer groups and the distribution of peak widths was plotted using the R package ggplot2 (43).

### Identification of 3D chromatin clusters using Hi-C or ChIA-PET data

Hi-C or POLR2A ChIA-PET BED files containing significant 3D interactions loops from HepG2, A549 and MCF7 cells were downloaded from GEO (Supplementary Table S1). Left and right arms of the chromosomal loops were separately intersected with active enhancers and TSSs using bedtools window under the default setting of 1 kb gap. Loops overlapping with the same enhancers or TSSs on either arm were further grouped into ‘clusters’, which were used in all the comparisons unless stated otherwise. For classification of clusters by enhancers, clusters were named non-exclusively as cSE-, dSE-, aSE- or rEh- containing clusters, respectively, as long as they overlap with at least one cSE, dSE, aSE or rEh. For each cluster, all the expressed genes (FPKM > 2) (48) whose TSSs overlap with that cluster were considered target genes of all the enhancers that overlap with the same cluster. Enhancer counts per cluster represented the number of unique enhancers within each cluster; Contacts per cluster represented the sum of all contacts detected from all the loops assigned to each cluster; Cluster span was calculated by subtracting max(end) by min(start) of all the loops for each cluster.

### Super high occupancy enhancer prediction

To determine factor signals that best predict super-high-occupancy enhancers (aSEs and dSEs), we first ranked all active enhancer peaks according to the factor signal value (highest to lowest). For any active enhancers where factor peaks were not present, the signal value was assigned zero. Ranked enhancer peaks were then annotated based on their enhancer class and a receiver-operating characteristic (ROC) curve was plotted to evaluate the factor signal ranking for its predictive performance in identifying aSEs and dSEs. To calculate the optimal, factor-specific signal cutoff for predicting aSEs and dSEs, we implemented the following: Active enhancers were ranked according to the factor signal value at that peak (lowest to highest), with enhancers lacking overlapping factor peaks assigned a signal value of zero. The ranked signal value was plotted and the global inflection point was defined as the value where a line tangent to that point has a slope equal to the maximum signal value minus the minimum signal value divided by the total number of peaks: (Signal_max_ – Signal_min_)/# peaks, similar to the ROSE method but without stitching. Next, we calculated a local inflection point for the lower portion of the curve, using the global inflection point as the new maximum value. Using this newly-defined cutoff, the true positive and false positive rates were calculated for each indicated factor.

### Visualization and statistical analysis

Individual ChIP-seq, GRO-seq, and STARR-seq signal profile plots and corresponding heatmaps were generated using the computeMatrix and plotHeatmap from the deepTools suite (49). Heatmaps depicting the normalized factor signal values for all factors across all individual enhancer peaks or summarized across all enhancer class peaks was generated using the ComplexHeatmap R package (50) and GraphPad Prism, respectively. Individual factor signal value normalization was conducted using min-max normalization. *P* values for all boxplots and violin plots were calculated based on the Wilcoxon signed-rank test using the pairwise.wilcox.test function from the R stats package. GraphPad Prism and BedTools2 were used to generate heatmaps.

## RESULTS

### Identification of enhancers bound by exceptionally high levels of transcription factors and co-regulatory proteins

**Figure 1.**
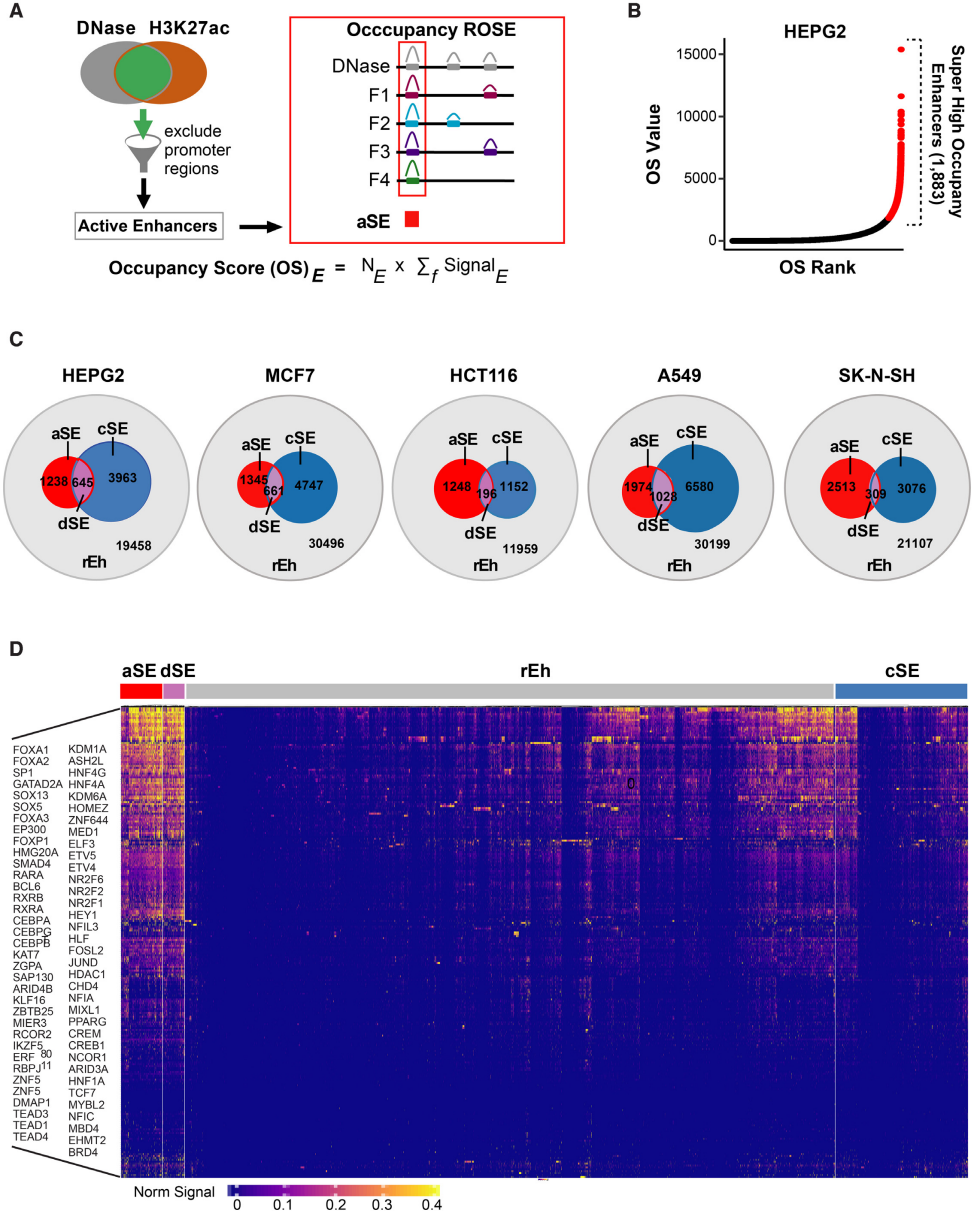
Re-classification of enhancers according to the overall occupancy levels of TFs and co-regulatory proteins. (**A**) Approach to calculate Occupancy Score (OS). For each active enhancer, an OS was calculated by multiplying the number of factors bound and the sum of factor signal values. (**B**) Plot of OS values and rankings across all active enhancers in HepG2 cells. All enhancers that surpass the cut-off for super-high occupancy enhancers are highlighted in red. (**C**) Venn diagrams comparing the overlaps between super-high occupancy enhancers defined by OS values in (B) and classic SEs defined by the standard ROSE method for the indicated cell lines. aSE: Super-high occupancy enhancers outside the classic SE regions; cSE: Enhancers within the classic SE regions but do not qualify as super-high occupancy enhancers; dSE: Super-high occupancy enhancers within classic SE regions; rEh: Enhancers outside the classic SE regions and do not qualify as super-high occupancy enhancers. (**D**) Heatmap displaying the normalized signal values for the indicated factors across all four re-classified active enhancer types in HepG2 cells.

Inspired by the Ranking Of Super Enhancer (ROSE) method (9), which is commonly used to define classic SEs by stitching together long stretches of enhancers followed by ranking the ChIP-seq signal intensities from one of the active enhancer markers (i.e. H3K27ac, H3K4m1, BRD4, p300 or MED1), we sought to unbiasedly identify super-high-occupancy enhancers by ranking all enhancers according to the combined signal intensity and genomic occupancy levels of all high-quality DNase-seq and ChIP-seq data from TFs and co-factors available through GEO in five widely-studied human cancer cell lines representing five different tumor types: HEPG2 (hepatocellular carcinoma cells), HCT116 (colorectal adenocarcinoma cells), A549 (non-small cell lung cancer adenocarcinoma cells), SK-N-SH (bone-marrow metastatic cells from a patient with neuroblastoma), and MCF7 (luminal breast cancer cells). To ensure uniformity, all non-ENCODE data were re-processed according to the highly stringent ENCODE pipeline and removed if they did not pass quality control measures (Supplementary Table S1, see methods). We called DNase-seq peaks demarcated by H3K27ac and located more than 2.5 kb from TSS as active enhancers (Figure 1A). To quantify the co-occupancy level of TFs and co-factors, we calculated the occupancy score (OS) by multiplying the number (N) of factors bound at each active enhancer (*E*) by the sum of normalized peak signals of these factors (*f)*. By ranking the active enhancers by their OS values, we identified a subset of enhancers with exceptionally high levels of factor binding, which represent between 5.7% and 11.3% of all active enhancers in the five cell lines examined (Figure 1B, C, Supplementary Figure S1A, Table S2).

Notably, in the five cell lines examined only 14–34% of the newly defined super-high-occupancy enhancers identified by our algorithm qualify as classic SEs based on the standard ROSE method (9) (Figure 1C). We hypothesized that these newly defined enhancers may represent a distinct class of SEs that function in parallel with classically defined SE to maintain oncogenic transcription. To enable head-to-head comparisons between super-high-occupancy enhancers and the classic SEs, we re-categorized all active enhancers into four classes: We termed super-high-occupancy enhancers not captured by the standard ROSE method as autonomous SEs (aSEs) to reflect their ability to recruit exceptionally high levels of factors independently, and conversely renamed classic SE that did not qualify as super-high-occupancy enhancers as constituent SEs (cSEs) given their proximal locations to one another and their cooperative nature in regulating transcription (8–10). Finally, we refer to SEs called by both methods as dual SEs (dSEs), and the remaining enhancers as rEhs. By this classification system, aSEs, cSEs, dSEs and rEhs represent 3.6–8.8%, 7.9–16.5%, 1.3–2.6% and 75.9–82.1% of all active enhancers, respectively (Supplementary Table S2).

Notably, compared to cSEs and rEhs, aSEs and dSEs are not only co-bound by significantly higher numbers of different factors, but the average signal values for most factors present at these sites are also significantly higher (Figure 1D, Supplementary Figure S1B, C), implying increased copies and/or binding affinity of individual factors. Considering the majority of super-high-high-occupancy enhancers were excluded from classic SE regions (i.e. aSEs) in all five cell lines (Figure 1C), we set out to compare the functional characteristics underlying the four subtypes of reclassified enhancers.

### The newly-identified autonomous super enhancers bear the functional hallmarks of classic super enhancers

**Figure 2.**
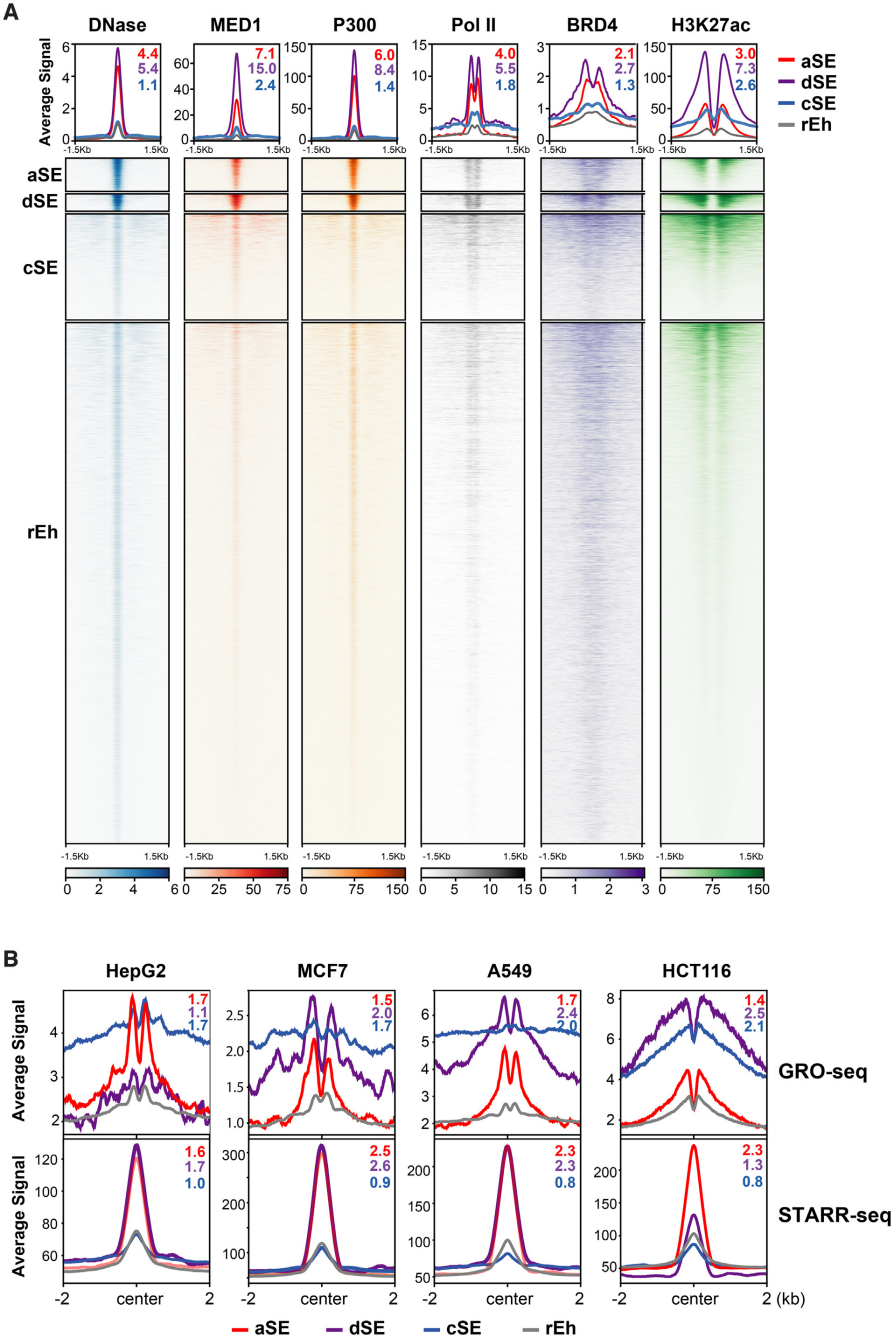
The newly-defined aSEs exhibit the functional hallmarks of super enhancers. (**A**) Heatmaps and density plots comparing DNase-seq and ChIP-seq signals from the indicated transcriptional co-activators and H3K27ac within ±1.5 kb of all four classes of enhancers in HepG2 cells. Values represent SE group signal fold enrichment over rEh. (**B**) Density plots of average GRO-seq (top) and STARR-seq (bottom) signals within ±2 kb of all four classes of enhancers in the indicated cell lines. Values represent SE group signal fold enrichment over rEh.

Classic SEs are known to exhibit exceptionally high levels of enrichment for P300, H3K27ac, BRD4, the mediator complex, and RNA Pol II (9–11). Indeed, all three newly classified SE subtypes exhibit significantly elevated MED1, P300, BRD4, H3K27ac and RNA Pol II signals compared to rEhs across all five cell lines examined (MED1: 2.4–15-fold; P300: 1.4–8.4-fold; BRD4: 1.3–2.7-fold; H3K27ac: 2.6–7.3-fold; RNA Pol II: 1.8–5.5-fold; *P* < 10^–10^ for all; Figure 2A and Supplementary Figure S2A).

Another key feature associated with classic SEs is their heightened ability to transcribe bi-directional, short-lived eRNA, which can be detected by GRO-seq (12,14). Analysis of GRO-seq data available for four of the cell lines included in our study revealed consistently elevated eRNA expression levels in both directions of the three newly classified SE subtypes relative to rEh (aSE: 1.4–1.7-fold; dSE: 1.1–2.5-fold; cSE: 1.7–2.1-fold; *P* < 10^–23^ for all; Figure 2B). Interestingly, although the eRNA signals sharply drop off to the baseline level within 1–2 kb from aSE, the decline in eRNA signals from cSE and dSE is much more gradual (Figure 2B), likely due to the accumulative eRNA signal from adjacent cSE and/or dSE. In agreement with this, aSE are located on average >1 kb from the most adjacent enhancer of any kind and >1 Mb from the closest classic SE regions, while cSE and dSE, which be definition exist within classic SE regions, are typically present within <1 kb of the closest enhancer (Supplementary Figures S2B, C).

High-throughput reporter assays such as self-transcribing active regulatory region sequencing (STARR-seq) have enabled quantification of individual enhancer activities across the entire genome (51). Comparison of STARR-seq signals among the four enhancer classes showed that relative to rEhs, individual aSEs and dSEs but not cSEs drive dramatically higher levels of transcription across the four cell lines examined (aSE: 1.6–2.3-fold, *P* < 10^–11^ for all lines; dSE: 1.7–2.6-fold, *P* < 10^–7^ for all lines except HCT116; cSE: 0.8–1.0-fold, *P* = 0.9–0.03; Figure 2B). There results re-enforce the notion that while cSEs require cooperation of adjacent enhancers to maintain heightened transcriptional activities that are lost when individual cSEs are placed in isolation in STARR reporter constructs, aSEs and dSEs are capable of establishing highly active, stand-alone transcriptional complexes at individual enhancer sites.

### Autonomous and dual super enhancers are anchored by a dense array of tightly-bound lineage-specific master transcription factors

Next, we investigated potential mechanisms that could explain how individual aSEs are able to anchor such a large number of TFs and co-activators. Previous analysis of HOT loci in individual cell lines suggest that higher GC contents may contribute to the increase in TF binding affinity at these sites (26,27,32). However, we did not detect a consistent increase in GC contents or CpG islands in aSE and dSE peaks compared to those for cSEs and rEhs (Supplementary Figure S3A-B), even though aSEs and dSEs associate with significantly broader and stronger DNase-seq peaks compared to cSEs and rEhs across the five cell lines (Figure 2A, Supplementary Figures S2A, S3C).

**Figure 3.**
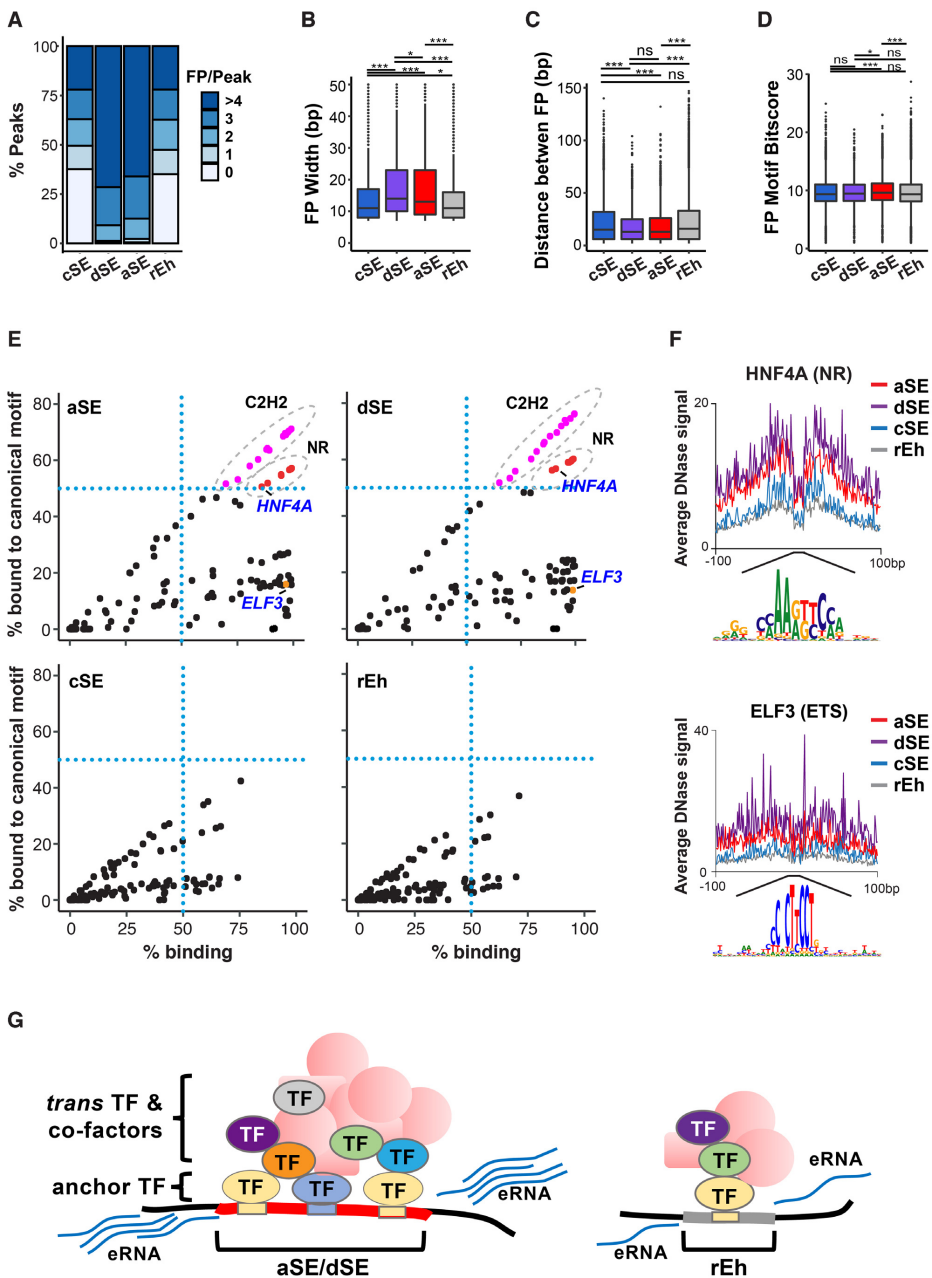
aSEs and dSEs are densely occupied by lineage-specific anchoring TFs. (**A**) Percent of enhancers within each enhancer class that contain the indicated number of DNase footprints per enhancer peak in HepG2 cells. (B−D) Box plot showing average DNase footprint width (**B**), distance between footprints (**C**), or motif bitscore (**D**) across all HepG2 enhancer classes. ns: not significant; *P< 0.05; ***P< 0.0005. All *P*-values determined using Wilcoxon rank sum test. (**E**) Plots showing percent of enhancer peaks bound by individual TFs as determined by ChIP-seq and percent of occupied peaks that contain DNase footprints matching to their canonical motif(s) at each enhancer class in HepG2 cells. Any TFs that bind to >50% of all peaks within the enhancer class AND whose motif are present more than 50% of the occupied peaks (upper right quadrant of blue dotted lines) are classified as anchoring TFs. Pink dots circled by grey dotted line all belong to C2H2 Zinc Finger family. Red dots circled by grey dotted line are all nuclear receptors (NR). Two representative anchoring (HNF4A) and non-anchoring (ELF3) TFs are marked in blue. (**F**) Density plots of average bias-corrected DNase-seq signals at ±100 bp from the center of the HNF4A (top) and ELF3 (bottom) footprints across all enhancer classes in HepG2 cells. (**G**) Schematic summarizing the differences in factor binding and eRNA transcription at aSE/dSE compared to rEh.

TFs making direct contacts with DNA are known to protect their bound-motifs from DNase cleavage, leaving so-called footprints within DNase hypersensitive peaks (44,52). Taking advantage of the newly published high-resolution TF footprint maps for HepG2, MCF7 and A549 cells by ENCODE (53,54), we compared the abundance, distribution and specificity of all of the identified TF footprints within the four types of enhancers. Even though there is no distinct difference in the overall number of known TF motifs per peak among the enhancer classes (Supplementary Figure S3D), aSE and dSE peaks on average contain significantly more footprints than cSE and rEh peaks (Figure 3A, Supplementary Figure S4A), indicating higher numbers of TFs directly binding to aSE and dSE compared to cSE and rEh. Moreover, the average width of the TF footprints within aSE and dSE peaks are considerably larger than those within cSE and rEh peaks (Figure 3B, Supplementary Figure S4B), whereas the distances between adjacent footprints are significantly smaller within aSE and dSE peaks relative to those within cSE and rEh peaks (Figure 3C, Supplementary Figure S4C). Finally, the TF footprints within aSEs and dSEs matched significantly better to their canonical DNA-binding motifs than those within cSEs and rEhs (Supplementary Figures 3D, S4D), which may also contribute to the increased binding specificity of anchoring TF at these sites.

Master lineage and oncogenic TF are known to be highly enriched at classic SEs (8,9,15,16). To test whether these TFs also contribute to the formation of stand-alone aSEs, we plotted the percentage of aSE peaks bound by each TF (as shown by ChIP-seq) against the percent of binding by the same TF at its canonical motif (as predicated by DNase footprinting) in HepG2 cells, which have the most available TF ChIP-seq data. Strikingly, among 151 TFs examined, 100 were detected at >50% of all aSEs peaks, with 26 binding to >95% of aSE peaks (Figure 3E, Supplementary Table S3). Importantly, bona fide hepatic lineage-specific TFs including members of the nuclear hormone receptor (NR) family (55), are not only among the most enriched TFs but also exhibit the highest percent of binding to their canonical motifs (Figure 3E-F, Supplementary Table S3), indicative their roles as anchoring TFs at aSEs. While very similar enrichment patterns were observed at dSE sites (Figure 3E), not a single TF is associated either directly or indirectly with >50% of cSEs or rEhs and meets the threshold of 50% binding to its canonical sites (Figure 3E).

To verify whether lineage-specific TFs are also responsible for anchoring aSEs and dSEs in other cell lines, we compared the frequency of DNASE footprints detected at each enhancer class in MCF7 and A549 cells. Similar to HepG2 cells, the C2H2 and NR motif families are the top most differentially enriched footprints at aSEs and dSEs compared to rEhs in MCF7 cells (Supplementary Figure S4E). In contrast, in KRAS mutant A549 cells, the bZIP motif family is the most differentially enriched footprint at aSEs and dSEs compared to cSEs and rEhs (Supplementary Figure S4E-F). Consistent with this observation, analysis of available A549 ChIP-seq data for several AP-1 TFs showed that together they occupy >99% of aSE and dSE, but only are present in <50% of cSE or rEh (Supplementary Figure S4G). Given the well-established role of the AP-1 transcriptional machinery as a core downstream effector of MAPK signaling (9,56–58), our results highlight the potential key involvement of aSEs and dSEs in KRAS-driven tumorigenesis.

In light of the lineage-specific footprint enrichment signatures at the three SE subtypes, we compared the specificity of enhancer classification across cell lines. Consistent with previous reports (59–62), large fractions of enhancers that met the criteria of cSEs in one cell line were either completely inactive or not classified as either cSEs or dSEs in other cell lines (Supplementary Figure S4H). Similarly, the majority of enhancers qualified as aSEs or dSEs in one cell line did not reach the cutoff of aSEs or dSEs in other cell lines, although, generally higher percentages of aSEs and dSEs remain at least as active enhancers in other cell lines as compared to cSEs and rEhs (aSE: 63%; dSE: 69%; cSE: 40%; rEh: 39%; Supplementary Figure S4H), consistent with an increased conservation PhastCon score at these sites (Supplementary Figure S4I).

Together, our analysis suggests that while all three SE subtypes likely operate in a lineage-specific manner, the highly conserved aSE and dSE sites tend to be directly and strongly bound by a dense array of master lineage TFs, which serve as anchors for subsequent recruitment of a large number of additional TFs and co-factors *in trans* (Figure 3G).

### Autonomous and dual super enhancers are enriched for both transcriptional co-activators and co-repressors

To gain further insights into the differences among the newly defined SE subtypes, we analyzed the binding frequency and signal strength of a broad spectrum of transcriptional co-factors and chromatin remodelers. Compared to cSEs, aSEs and dSEs exhibited significantly higher binding by MED1, P300 and other transcriptional co-activators in terms of both overall percentages and signal intensity (MED1: 1.9–2.8-fold enrichment over cSE; P300: 4.3–5.4-fold enrichment over cSE; Supplementary Figures S5A, B). In HepG2 cells, the co-activators H3K27 demethylase KDM6A, H3K16 acetyltransferase KAT7, H4K16 acetyltransferase KAT8, and H3K4 methyltransferase complex subunit ASH2L were detected at over 89%, 60%, 90% and 95% of aSE and dSE regions compared to <22%, 19%, 32% and 38% of cSEs, respectively (Supplementary Figure S5A). Similarly, in HCT116 cells the H3K4 methyltransferase KMT2D is present in 90% of aSE and 93% of dSEs versus 49% of cSEs (Supplementary Figure S5B). Interestingly, transcriptional co-repressors, including components of the SIN3A and NCoR co-repressor complexes, are also enriched at aSEs and dSEs relative to cSEs and rEhs in all five cell lines (Supplementary Figures S5A, B). The heightened co-occupancy by both transcriptional co-activators and co-repressors at aSEs and dSEs indicate that their transcriptional activities are subjected to complex regulation. In agreement with this theory, despite the clear increase in occupancy by multiple acetyltransferases and H3K4 methyltransferases at aSEs and dSEs compared to cSEs (Supplementary Figures S5A, B), all three SE subtypes displayed similar signal values for activating histone marks such as H3K4me1, H3K9ac, H3K4me2 and H3K27ac (Supplementary Figure S5C). These results highlight the importance of dissecting how the dynamic interactions between individual co-activator and co-repressor complexes modulate overall SE activity.

### Autonomous and dual super enhancers engage in cohesin-mediated long-distance interactions and promote the high expression of distal target genes

**Figure 4.**
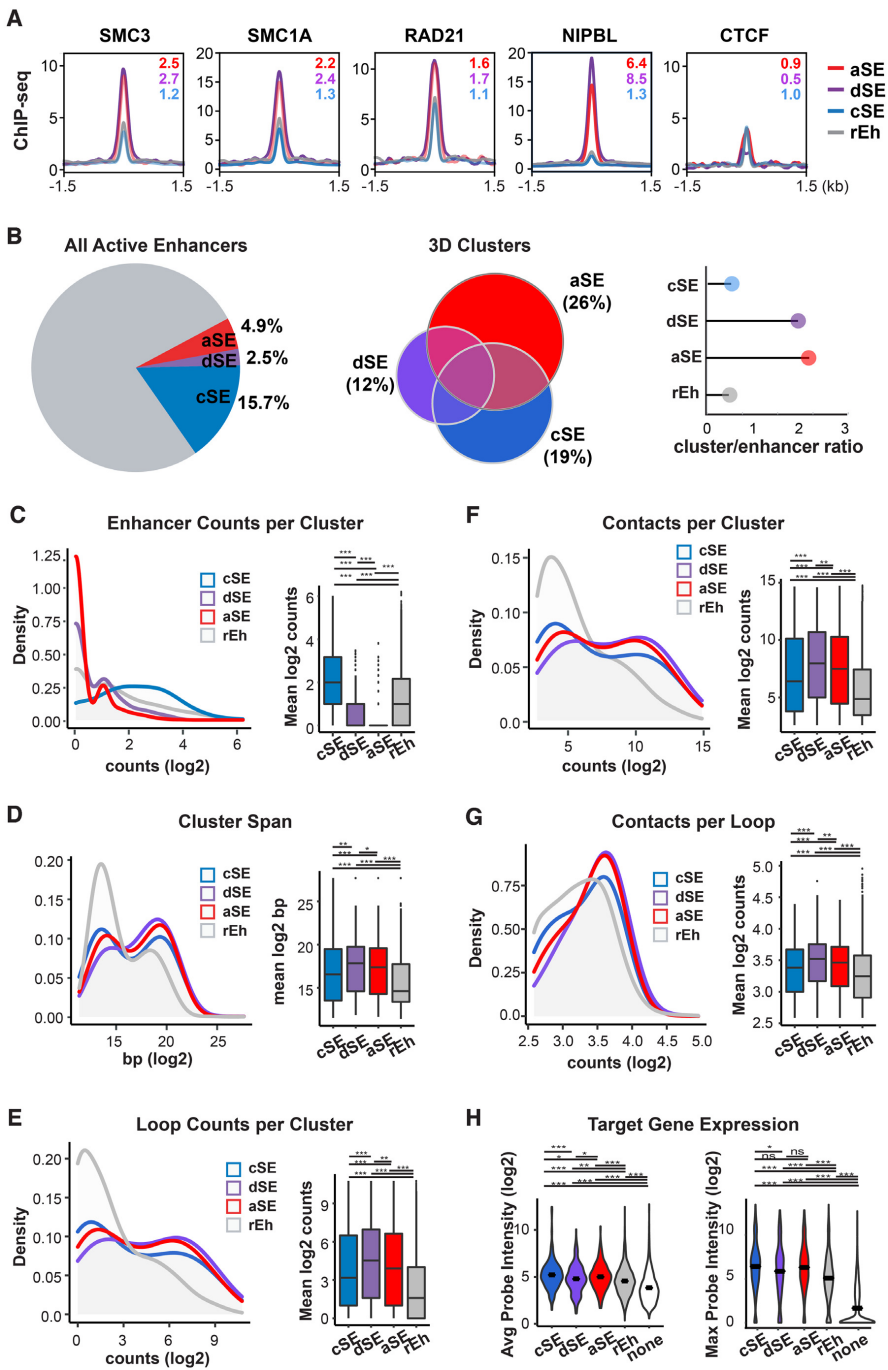
aSEs and dSEs engage in cohesin-mediated long-distance interactions and promote the high expression of distal target genes. (**A**) Density plots of ChIP-seq signals from the indicated cohesin-associated factors at all enhancer classes in HepG2 cells. Values represent SE group signal fold enrichment over rEh. (**B**) Pie chart (left) displaying the percent of active enhancers belonging to each SE subtype from Figure 1C, euler diagram (middle) displaying the percent of 3D chromatin clusters containing each SE subtype based on Hi-C data, and lollipop plot (right) displaying the ratio of 3D cluster count to enhancer count for each SE subtype in HepG2 cells. (**C**−**G**) Corresponding density (left) and box (right) plots of indicated parameters of 3D chromatin clusters containing each SE class based on Hi-C data from HepG2 cells. ns: not significant; *P< 0.05; **P< 0.005. ***P< 0.0005. All *P*-values determined using Wilcoxon rank sum test. (**H**) Violin plots of the expression levels of all genes (left) or the highest expressed gene (right) within each Hi-C cluster across all Hi-C clusters containing each SE class in HepG2 cells. Bars represent mean value. ns: not significant; **P< 0.005. ***P< 0.0005. All *P*-values determined using Wilcoxon rank sum test.

Cohesin is a conserved, ring-like complex whose core components consist of SMC1A, SMC3, RAD21 and STAG1/2, and is recruited to accessible genomic loci by the NIPBL-MAU2 cohesin-loading complex (9,63–65). Through interactions with mediator and master TF at non-CTCF sites, cohesin has also been shown to stabilize chromatin looping and long-distance enhancer-promoter interactions at highly transcribed genes (19,66–70). Strikingly, in all the cell lines with cohesin data available, aSEs and dSEs were preferentially bound by the cohesin-loading factor NIPBL and other core cohesin subunits such as RAD21, SMC1A, and SMC3, while simultaneously replete of CTCF (SMC3: 4.5–5.1-fold enrichment over rEh; SMC1A: 3.3–3.9-fold enrichment over rEh; RAD21: 2.8–3-fold enrichment over rEh; NIPBL: 13.4–18.1-fold enrichment over rEh; CTCF: 0.9–1.6-fold enrichment over rEh; Figure 4A, Supplementary Figure S6A). In contrast, cSEs and rEhs displayed much less binding by NIBPL and other cohesion subunits (Figure 4A, Supplementary Figure S6A).

This intriguing observation prompted us to examine how individual types of enhancers make contacts with other genomic loci utilizing existing 3D chromatin interaction data. Recent studies indicate that chromatin loops often form hyper-connected clusters, and enhancers within the same clusters tend to function in unison to co-regulate target genes located within the same clusters (71,72). Based on these reports, we first identified high-confidence chromatin loops detected by either high throughput chromatin conformation capture (Hi-C: HepG2 and A549) or POL2RA chromatin interaction analysis with paired-end tag sequencing (ChIA-PET: MCF7), and grouped together any chromatin loops with one or both arms located within 1 kb of a common set of TSS and/or enhancers, essentially collapsing a network of inter-connected chromatin loops into a single cluster (see methods). Strikingly, even though aSEs and dSEs combined represent <8% of all enhancers, they are present in nearly half of all enhancer-containing clusters (Figure 4B, Supplementary Figure S6B). In contrast, cSEs, which encompasses 13–17% of all enhancers, are only present in 25–32% of all clusters (Figure 4B, Supplementary Figure S6B). Another major difference is how frequently each enhancer class co-occurs with enhancers of the same type in each cluster that they belong to: Whereas a large majority of aSEs and dSEs are present in clusters with very low aSE or dSE enhancer counts, respectively, cSEs are enriched in clusters with very high cSE enhancer counts (Figure 4C, Supplementary Figure S6C). Nevertheless, similar to clusters that contain cSEs and/or dSEs, aSE-occupied clusters span significantly longer distance, contain significantly more loops per cluster, and form significantly more contacts per cluster than clusters occupied by rEhs (Figure 4D−F, Supplementary Figure S6D-F). At the individual loop level, SE-occupied loop arms, regardless of subtypes, also consistently make stronger contacts with the opposite arms (Figure 4G, Supplementary Figure S6G). Intriguingly, among cSE-occupied clusters or loops, the major also contain aSEs or dSEs and form significantly more contacts than those without aSEs or dSEs (Supplementary Figure S6H-I), suggesting that aSEs and dSEs may play dominant roles in stabilizing long-range chromatin interactions.

Comparisons POL2RA ChIA-PET signal at enhancer sites revealed that all SE subtypes, exhibit substantially more POL2-associated interactions compared to rEh sites in MCF7 cells (Supplementary Figure S7A). Correspondingly, genes located within the same clusters as any of the three SE subtypes are expressed at significantly higher levels than genes clustered with rEhs or no distal enhancers in all the cell lines examined (Figure 4H, Supplementary Figure S7B, C). Among genes regulated by cSE-containing clusters, expression levels are generally higher when the clusters are co-occupied by aSEs and/or dSEs compared to those without aSEs and dSEs (Supplementary Figure S7D−F), suggesting that the presence of aSEs and dSEs further boosts the transcription activities of cSE clusters. Finally, RAD21-depletion significantly reduced the transcriptional output from genes that cluster with cSEs, dSEs or aSEs but had no or opposite effects on the transcription of genes associated with rEhs or no enhancers, and on eRNA production at enhancer sites (Supplementary Figure S7G).

**Figure 5.**
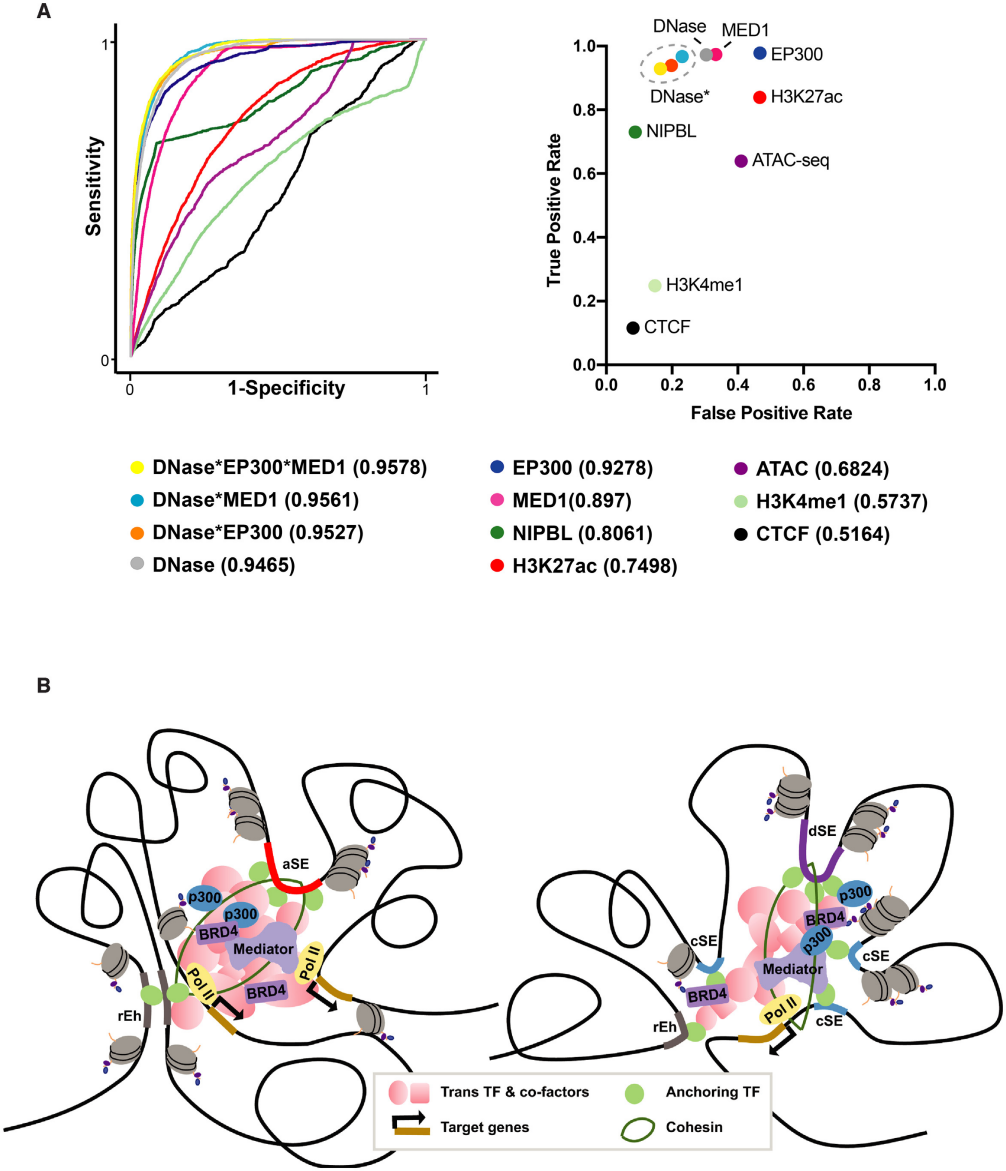
DNase, Mediator, and P300 Signals are highly effective predictive marks for aSEs and dSEs. (**A**) Receiver Operating Characteristic (ROC) curves displaying the predictive sensitivity and specificity (left) and scatter plot showing the true positive and false positive rates of predictions based on signal rankings (right) of the indicated chromatin accessibility data or ChIP-seq data from transcriptional co-activators and histone marks alone or in combination. Cutoff values for enhancer predictions were determined by ranking all active enhancers by the signal values to calculate the first inflection point of the curve, followed by calculating a second inflection point (cutoff value) of the bottom half of the curve using the first inflection point as the maximum value. (**B**) Working model displaying unique features in the 3D chromatin clusters associated with aSE and dSE. Our analysis suggests that aSE and dSE are broad accessible regions that serve as major anchoring points for lineage TFs, cohesin complexes and other co-factors, through which engage in extensive long-distance interactions with other enhancer and promoters and promote the transcription of highly-expressed genes.

Together, these results strongly support the notion that whereas cSE require the cooperative actions of proximally located other cSEs or dSEs to establish long-distance contacts, stand-alone aSE are capable of anchoring a large number of TFs, co-factors and the cohesin complex *in trans*, through which engage in highly robust, focal long-range genomic interactions to sustain the transcription of distal target genes (Figure 5B).

### Autonomous and dual super enhancers can be reliably predicted using DNase-seq and ChIP-seq data from Mediator and P300

By integrating a large number of chromatin accessibility, ChIP-seq and other types of genomic datasets, we have identified a new class of super-high-occupancy enhancers that possess all the functional hallmarks of SEs but falls outside the classically defined SE regions. Given that the availability of chromatin accessibility or ChIP-seq datasets is limited for most cell lines and tissues, it is important to develop a new prediction method for super-high-occupancy SEs that does not require a large number of datasets. To this end, we ranked all four types of enhancers from HepG2 cells according to their signals in DNase-seq, ATAC-seq or ChIP-seq from several commonly studied factors, assessed the sensitivity and specificity of each dataset in predicting aSEs and dSEs. Area Under the Curve (AUC) analysis revealed that signals from DNase-seq or ChIP-seq of P300 or MED1 achieved an AUC >0.89, corresponding to a >90% true positive rate for identifying aSEs and dSEs (Figure 5A). In contrast, CTCF, which has minimal overlap with aSE and dSE peaks, showed a poor predictive value with an AUC of 0.52 and <10% true positive rate (Figure 5A). Additionally, ranking active enhancers by ATAC-seq signals or active enhancer histone marks H3K27ac and H3K4me1 also proved to be less predictive of aSEs and dSEs compared to DNase, P300 and MED1 signals (Figure 5A). These findings are consistent with our earlier observation of specific enrichment of DNase, P300, and MED1 but not active histone marks at aSEs and dSEs relative to cSEs and rEhs (Figure 2A, Supplementary Figure S2A). Finally, we showed that combining DNase with MED1 or P300 further reduced the false discovery rate from >30% to ∼20% (Figure 5A), suggesting that in cells or tissues where the numbers of ChIP-seq data are limited, at aSEs and dSEs can be reliably identified using DNase-seq data combined with a single ChIP-seq data against MED1 or P300.

## DISCUSSION

First proposed in 2012, the concept of SE has become widely adopted into the scientific community, with roughly 400 publications and counting implicating SE in regulation of many important biological processes including embryonic development, cell differentiation, tumorigenesis, and therapy resistance. SE are classically defined as long stretches of active enhancers that are physically tethered together by multivalent, mega transcriptional complexes composed of numerous TFs and co-factors, which promote the transcription of distally localized lineage-specific genes through chromatin looping. Taking advantage of the large number of published ChIP-seq data from five commonly-used cancer cell of distinct lineages, we ranked enhancers based on both the numbers of factors bound and the relative signal intensities from each factor, and identified a set of enhancers meet all the functional criteria of SEs but are not captured by the widely adopted ROSE method. We termed these enhancers aSEs based on their ability to independently recruit many TFs and assemble mega transcriptional complexes *in trans*. Among classic SEs identified by the ROSE method, we re-classified those that also met the criteria of aSEs as dSE, while renaming the rest as cSE. Notably, as dSEs represent overlaps between our newly identified super-high-occupancy enhancers and the classically defined SEs, they were considered a separate enhancer class for comparison purposes in this initial study. However, given that dSEs share most if not all the physical and functional characteristics of aSEs, they could be considered a subset of aSEs in future studies.

Our in-depth analysis revealed that compared to cSEs and rEhs, aSEs and dSEs display dramatically higher levels of chromatin accessibility, as well as binding by TFs, MED1, P300 and other co-activators (Figure 2, Supplementary Figure S2). Among the three subtypes of SEs, dSEs exhibit the highest levels of chromatin accessibility and co-activator bindings (Figures 2, Supplementary Figure S2), suggesting that their proximity to other cSEs and/or dSEs could further boost the assembly of mega transcriptional complexes at these sites. Interestingly, relative to cSEs, aSEs and dSEs also exhibit increased binding by transcriptional co-repressors including several HDAC-containing histone deacetylase complexes (Supplementary Figure S5). A recent study by Gryder *et al.* showed that the recruitment of HDACs to SE is necessary for preventing aberrant ‘spreading’ of H3K27ac to adjacent loci, which is critical for maintaining proper 3D chromatin architecture and enhancer-promoter contacts(1,73–77). Additionally, we observed increased enrichment of chromatin remodeling proteins at aSE and dSE regions (Supplementary Figure S5). In particular, multiple components of the cohesin complexes are strongly enriched at aSE and dSE sites (Figure 4, Supplementary Figure S5). As cohesin has a well-established role in chromatin organization through DNA loop extrusion, it is plausible to assume cohesin complexes enriched at aSEs and dSEs play a direct role in facilitating their long-distance interactions. Indeed, we found that despite representing < 8% percentage of all enhancers, aSEs and dSEs are disproportionally involved in nearly 50% of all chromatin interactions (Figure 4B, Supplementary Figure S6B). Moreover, cSE-containing chromatin clusters that include dSEs or aSEs make significantly stronger contacts compared to those without dSE/aSE and are associated with higher target gene expression (Supplementary Figures S6I, S7D−F), suggesting that dSE/aSE may serve primary anchoring sites for long-distance interactions that involve cSE. Finally, we showed that silencing of RAD21 significantly reduced the transcriptional outputs from genes contacted by all three SE subtypes (Supplementary Figure S7G), implying a functional role of the cohesin complex in promoting transcription of SE-regulated genes. While further study is necessary to determine how cohesin is recruited and trapped at dSE/aSE, a recent study showed that the yeast cohesin complexes undergo phase separation in the presence of long-stretches of DNA (69), hinting that the cohesin complexes may be attracted and retained through multi-valent interactions with the large accessible clusters of DNA tethered together by mega transcriptional complexes assembled at SE sites.

Phase-separation has been increasingly recognized as a key mechanism in concentrating a large number of SE and co-factors and facilitating in long-distance interactions at classically defined SE sites (16,66,78). Transient phase-separated 1–2 MDa transcriptional complexes have been shown to assemble *in trans* at specific chromatin ‘hot spot’ regions in response to certain stimuli (79–81). The phenomenon has been elegantly demonstrated in estrogen-stimulated MCF7 breast cancer cells, where DNA-bound estrogen receptor α (ERα) initiates the assembly of the so-called MegaTrans complexes comprised of a large number of TF, co-activators, RNA Pol II, eRNA, and ribonucleoproteins, all bound in *trans* (66,80). Given the extraordinarily high density of factors present at aSE (Figures 1 and 2, Supplementary Figures S1−S3), it is reasonable to postulate that concentration-driven phase separation may occur at aSEs, similar to what has been observed at classic SE and MegaTrans sites (16,66). Indeed, aSE/dSE-enriched factors including MED1, RNA Pol II and cohesin have been shown to form concentration-dependent phase separated droplets in vitro or in vivo (69,80,82,83). Interestingly, in MCF7 cells where MegaTrans complexes were mostly studied, aSEs share very little overlap with MegaTrans hotspots (not shown), consistent with the fact that unlike MegaTrans complexes, aSE peaks are stable, conserved, highly accessible genomic regions typically anchored by more than three lineage TF as shown through DNase footprinting (Figure 3, Supplementary Figure S4). It is worth noting, however, ChIP-seq experiments capture the average signals of the entire cell population. Further studies using single cell genomics and other advanced imaging techniques will be extremely useful in establishing cellular heterogeneity and the dynamics of the transcriptional complex assembly and disassembly at aSE and dSE sites.

By integrating 3D chromatin interaction data with RNAseq, we showed that similar to cSEs and dSEs, aSEs are involved in extensive, highly robust long-distance interactions with highly transcribed genes (Figure 4, Supplementary Figure S6). Additionally, cSE-containing clusters may depend on the presence of aSEs and/or dSEs to sustain long-distance interactions and high transcriptional activity at target genes (Supplementary Figures S6, S7), strengthening the evidence that aSEs and dSEs are critical components of the regulatory architecture and therefore may play important roles in cancer cell survival. Classic SEs, which are highly enriched for BRD4, show heightened sensitivity to BET inhibitors (34). In contrast, aSEs show variable BRD4 binding (Supplementary Figure S2A) and therefore may not exhibit the same levels of sensitivity to BET inhibitors. Therefore, it could be of high therapeutic relevance to identify means to either co-target all SEs or selectively disrupt transcription programs driven by aSEs.

In summary, this study provides evidentiary support for expanding the classification of SEs to include stand-alone aSE and exposes a number of unique traits associated with this newly defined SE subclass. We demonstrate that aSE can be reliably identified by ranking the signal levels from DNase-seq alone or in combination with ChIP-seq data for MED1 or p300 (Figure 5A). Moving forward, this strategy can be combined with the classic ROSE method to better catalogue highly active, functionally dominant enhancers.

## DATA AVAILABILITY

All GEO and SRA datasets used throughout this paper can be found in Supplementary Table S1.

## Supplementary Material

gkab1105_Supplemental_Files
