# Supplementary material for: Master lineage transcription factors anchor trans mega transcriptional complexes at highly accessible enhancer sites to promote long-range chromatin clustering and transcription of distal target genes
Source: Nucleic Acids Res. 2021 Nov 24;49(21):12196–210. doi: 10.1093/nar/gkab1105 (PMC8643643; doi:10.1093/nar/gkab1105)
Supplement: gkab1105_Supplemental_Files [file gkab1105_supplemental_files.zip › TransSE_Figure_legends_suppl.pdf]

SUPPLEMENTARY FIGURE LEGENDS

Figure S1. Re-classification of enhancers according to the overall occupancy levels of TFs and co-regulatory proteins. Related to Figure 1.

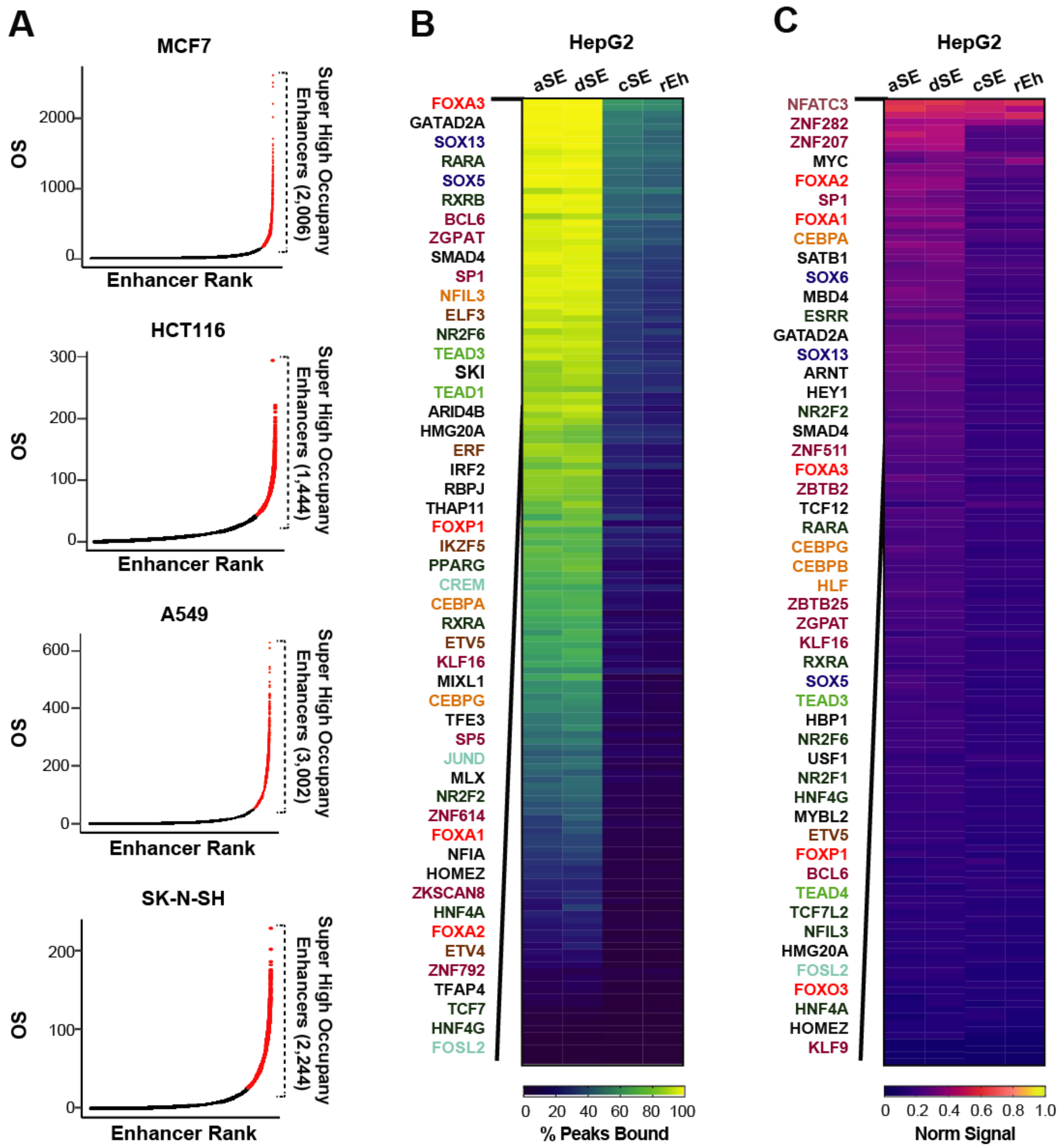

(A) Plot of OS values and rankings across all active enhancers in the indicated cell lines. All enhancers that surpass the cut-off for super-high occupancy enhancers are highlighted in red.

(B-C) Heatmap displaying the percent of peaks bound **(B)** and the average normalized signals **(C)** by individual TF among four classes of enhancers in HepG2 cells. TF belonging to Foxhead family are highlighted in red, C2H2 Zinc Finger family in purple, bHLH in brown, nuclear receptors (NR) in dark green, AP-1 in light blue and TEAD in green.

Figure S2. Comparisons of active transcription markers and distances between enhancers. Related to Figure 2.

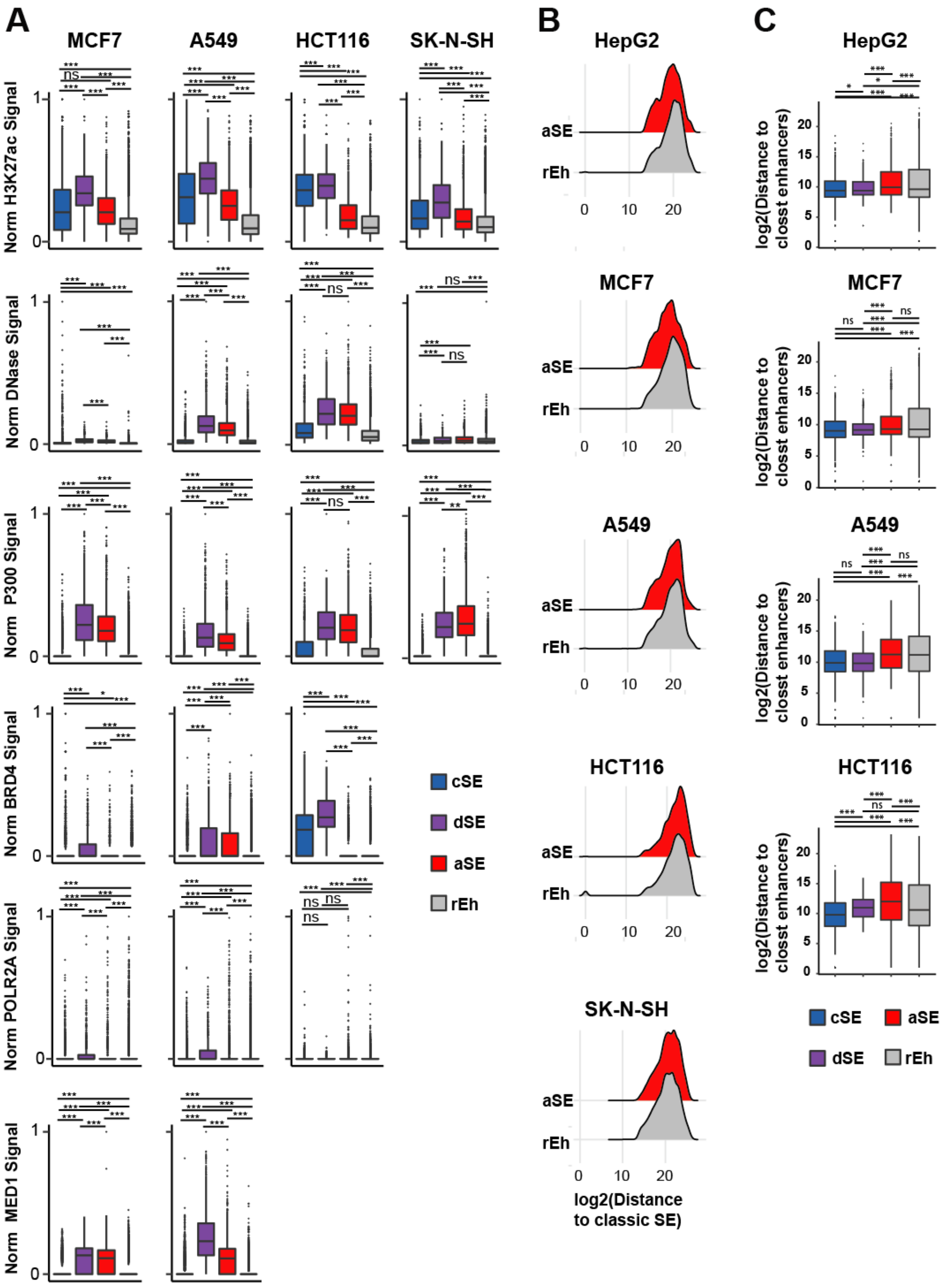

(A) Box plots showing average signals from DNase-seq and ChIP-seq from the indicated co-activator across all four enhancer classes from the indicated cell lines. ns: not significant; *$P<0.05$; **$P<0.005$; ***$P<0.0005$. All p-values determined using Wilcoxon rank sum test.

(B) Density plots displaying the distances from aSE and rEh to the closest classically-defined SE regions in the indicated cell lines.

(C) Box plots showing the average distance to the most adjacent enhancer from each enhancer type in the indicated cell lines. ns: not significant; *$P<0.05$; ***$P<0.0005$. All p-values determined using Wilcoxon rank sum test.

Figure S3. Comparisons of the physical characteristics of enhancer subtypes. Related to Figure 3.

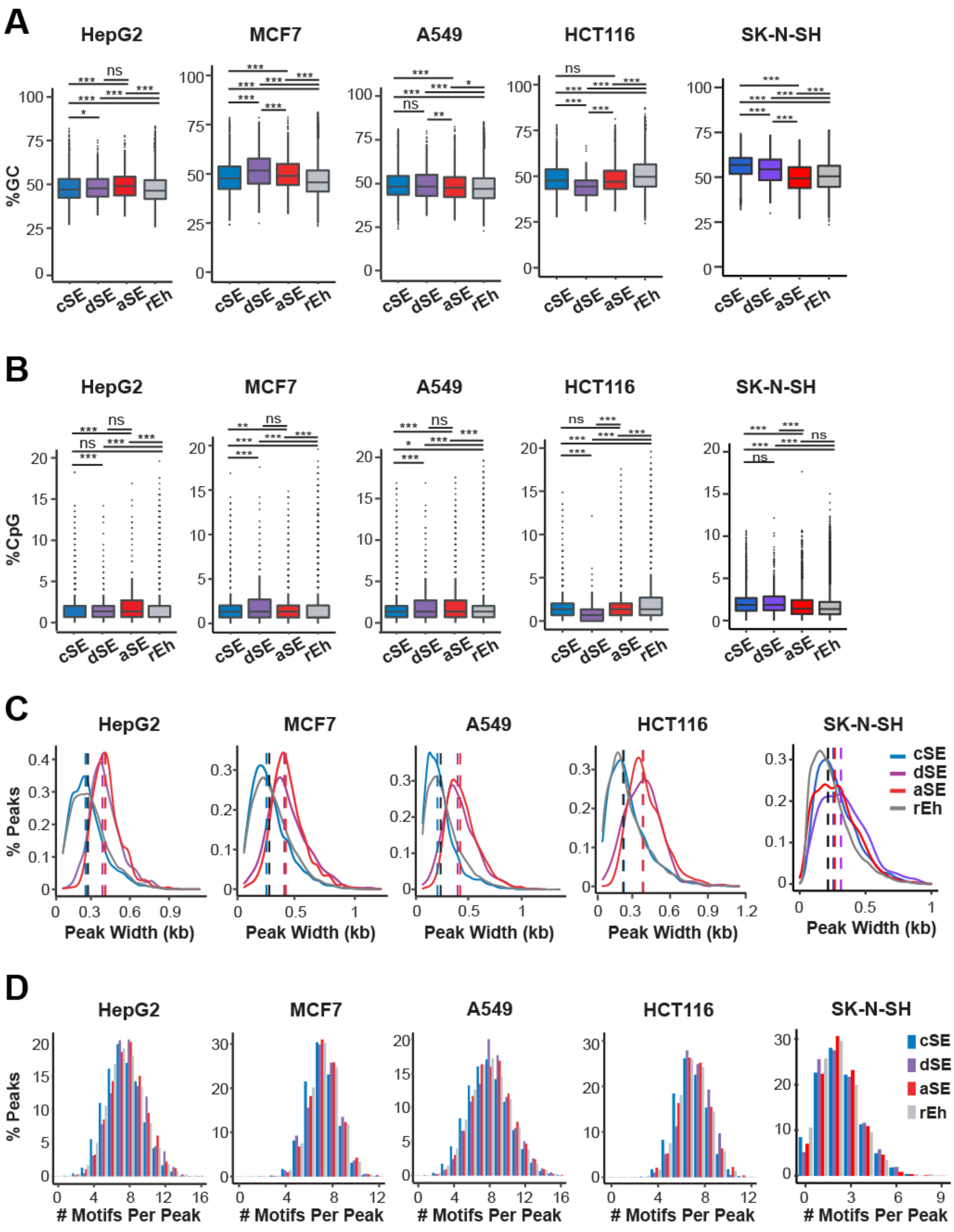

(A-B) Box plots showing GC **(A)** or CpG **(B)** percentages across all enhancer classes in the indicated cell lines.

(C) Distribution of peak widths for each enhancer class in the indicated cell lines. DNase-seq peaks were re-called with variable width setting to observe the naturally occurring differences in chromatin accessibility. HepG2 median widths: aSE – 410bp, dSE – 390bp, cSE – 259bp, rEh – 277bp. MCF7 median widths: aSE – 427bp, dSE – 416bp, cSE – 267bp, rEh – 291bp. A549 median widths: aSE – 426bp, dSE – 402bp, cSE – 214bp, rEh –246bp. HCT116 median widths: aSE – 388bp, dSE – 382.5bp, cSE – 233bp, rEh – 229bp. SK-N-SH median widths: aSE – 265bp, dSE – 313bp, cSE – 254bp, rEh –214bp.

(D) Distribution of the number of JASPAR motif clusters present per peak across all enhancer classes in the indicated cell lines.

Figure S4. aSEs and dSEs are conserved active enhancers, densely occupied by lineage-specific anchoring TFs. Related to Figure 3.

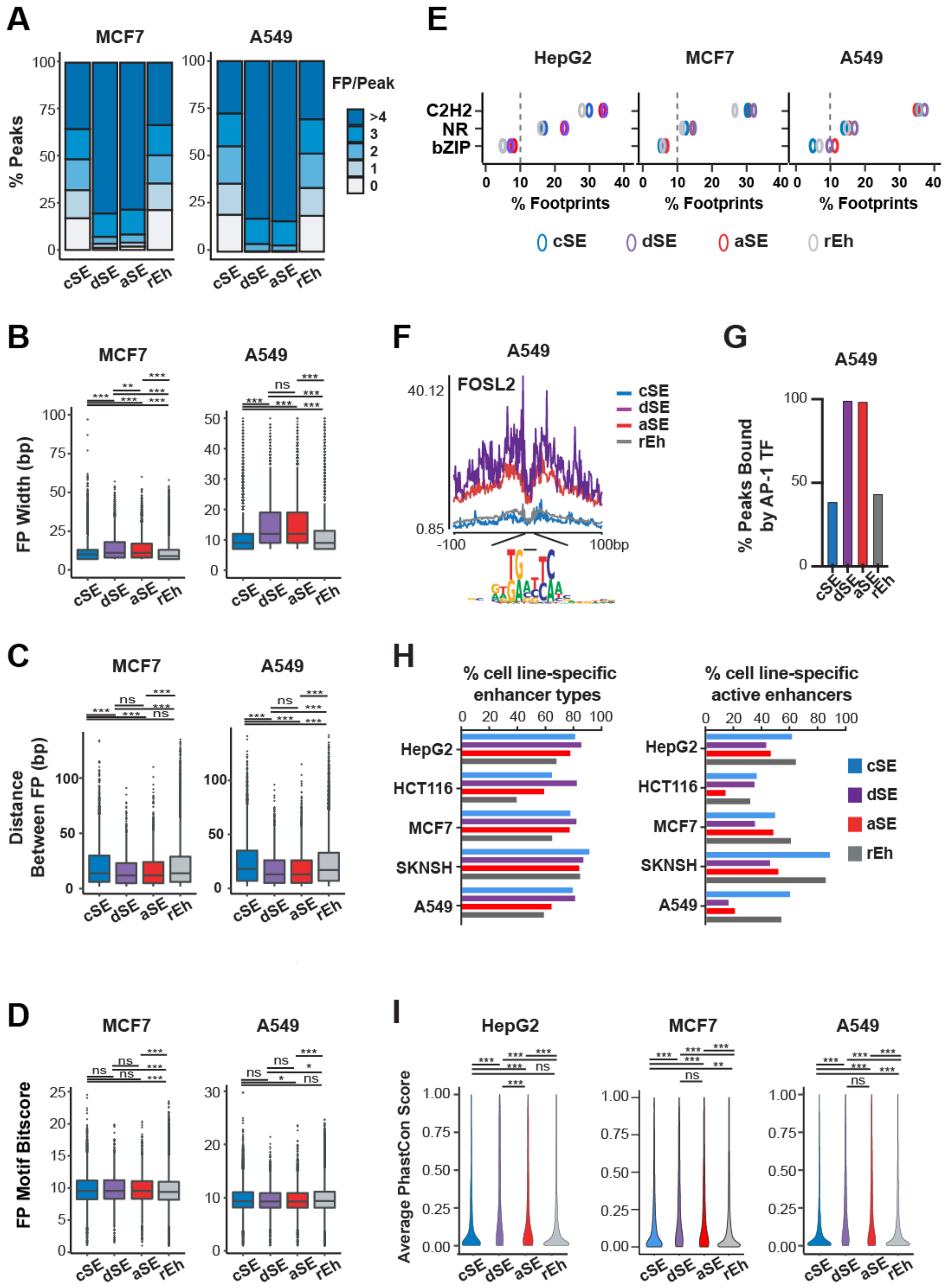

(A) Percent of enhancers within each enhancer class that contain the indicated number of DNase footprints per enhancer peak in MCF7 and A549 cells.

(B-D) Box plot showing average DNase footprint width **(B)**, distance between footprints **(C)**, or motif bitscore **(D)** across all enhancer classes in MCF7 and A549 cells. ns: not significant; *P<0.05; ***P<0.0005. All p-values determined using Wilcoxon rank sum test.

(E) The top 3 most differentially enriched motif family across all enhancer classes in HepG2, MCF7, and A549 cells.

(F) Density plots of average bias-corrected DNase-seq signals at +/-100bp from the center of FOSL2 footprints across all enhancer classes in A549 cells.

(G) Percent of enhancers from each enhancer class that overlap ChIP-seq peaks from at least one member of the AP-1 TF family in A549 cells. Analysis included FOSL2, ATF3, c-JUN, JUNB, and JUND ChIP-seq data.

(H) Bar graphs showing the percent of enhancers from each enhancer class in the indicated cell line that did not classify as the same enhancer class (left) or active enhancers of any type (right) in any of the other four cell lines examined.

(I) Box plot showing the conservation (PhastCon) score of each enhancer class in HepG2, MCF7, and A549 cells. ns: not significant; **P<0.005; ***P<0.0005. All p-values determined using Wilcoxon rank sum test.

Figure S5. aSEs and dSEs are co-occupied by transcriptional co-activators and co-repressors. Related to Figure 3.

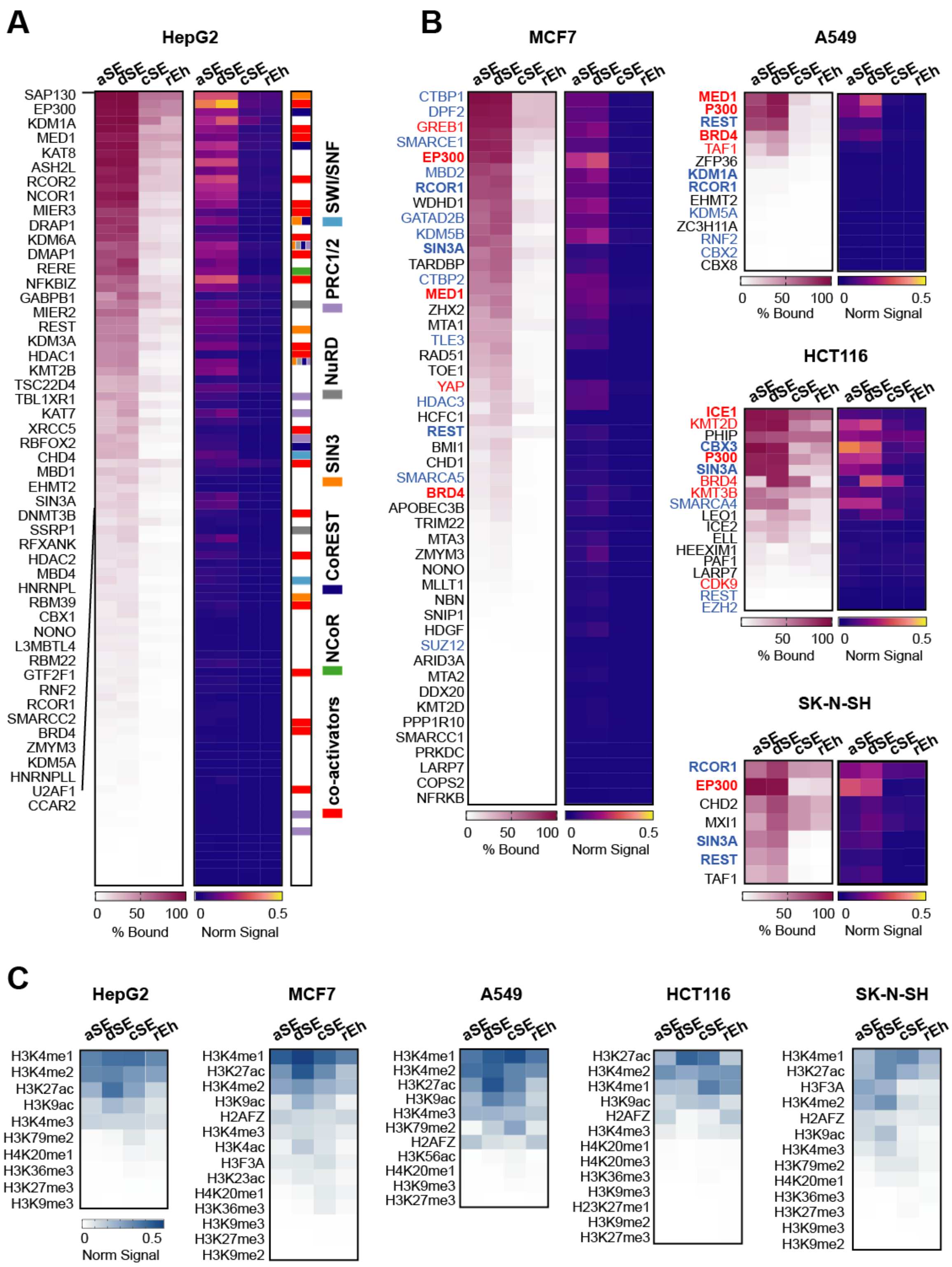

(A-B) Heatmap displaying the percent of peaks bound (left) and the average normalized signals (right) by individual co-factors among four enhancer classes in the indicated cell lines. Red bars mark known co-activators. Light blue bars and font colors mark components of the SWI/SNF complex. Green, dark blue, brown, grey and purple bars and font colors mark components of the NCoR, CoREST, SIN3, NuRD and PRC1/2 repressive complexes, respectively.

(C) Heatmap displaying the average normalized signals of indicated histone marks among four enhancer classes in the indicated cell lines.

Figure S6. aSEs and dSEs engage in cohesin-mediated long-distance interactions. Related to Figure 4.

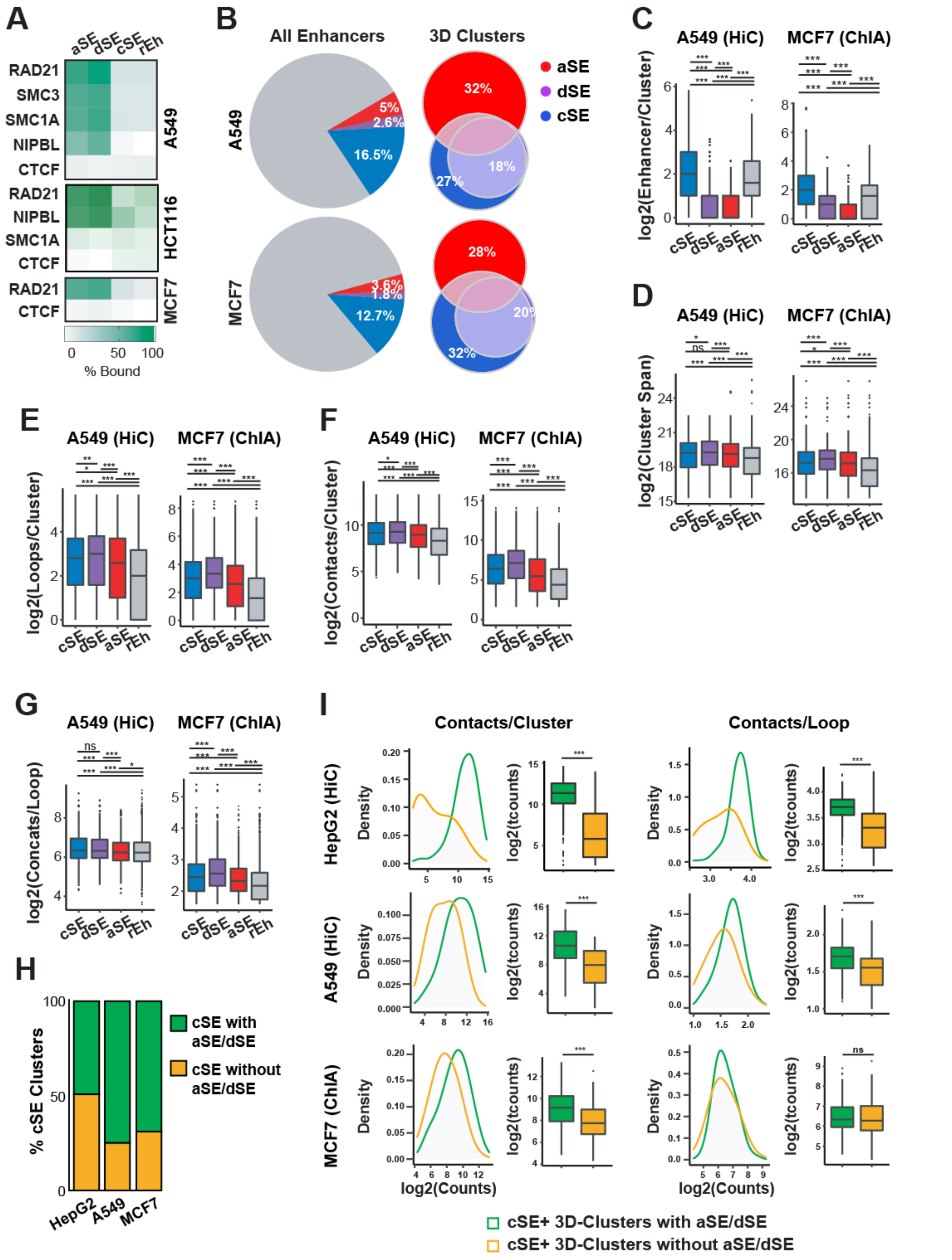

(A) Heatmap displaying the percent of enhancers within each enhancer class bound by the indicated cohesin-associated factors in the indicated cell lines.

(B) Pie charts (left) displaying the percent of active enhancers in A549 (top) and MCF7 (bottom) cells belonging to each SE subtype from Figure 1C, euler diagram (middle) displaying the percent of 3D chromatin clusters containing each SE subtype based on A549 Hi-C and MCF7PolII ChIA-PET data, and lollipop plots (right) displaying the ratios of 3D cluster count to enhancer count for each SE subtype.

(C-G) Corresponding density (left) and box (right) plots of indicated parameters of 3D chromatin clusters containing each SE class based on A549 Hi-C and MCF7 PolII ChIA-PET data. ns: not significant; **P*<0.05; ***P*<0.005. ****P*<0.0005. All p-values determined using Wilcoxon rank sum test.

(H) Stacked bar plot displaying the percentage of cSE-containing clusters with or without aSE and/or dSE.

(I) Corresponding density (left) and box (right) plots of indicated parameters comparing cSE-containing chromatin clusters with or without aSE/dSE based on the indicated 3D chromatin interaction data. ns: not significant; ****P*<0.0005. All p-values determined using Student's t-test.

Figure S7. All three subtypes of SEs promote the high expression of distal target genes in a Cohesin-dependent manner. Related to Figure 4.

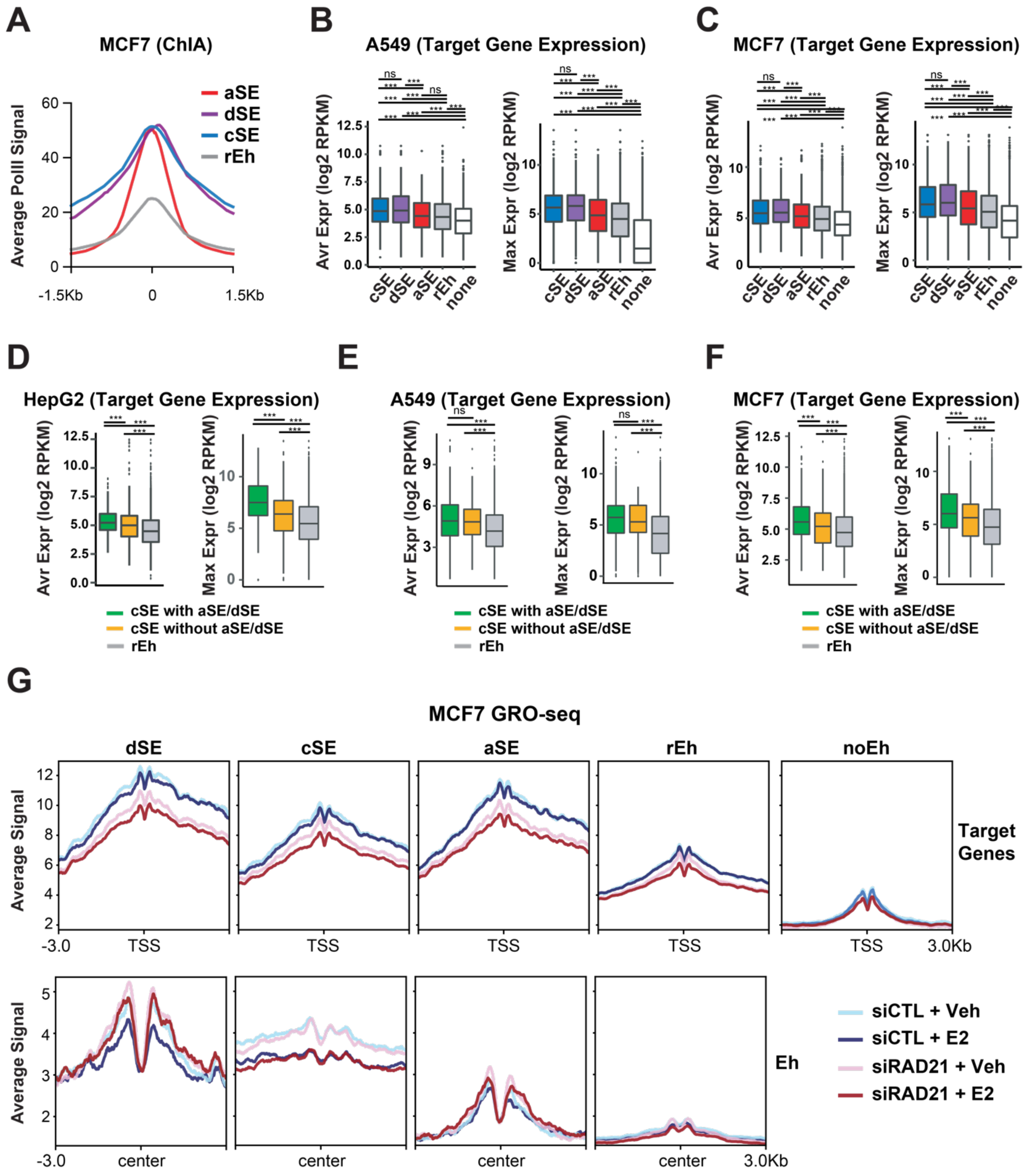

(A) Density plot of average POL2RA ChIA-PET signals at each enhancer class in MCF7 cells.

(B-C) Box plots of the expression levels of all genes (left) or the highest expressed gene (right) within each Hi-C cluster across all 3D chromatin clusters containing each SE class detected by Hi-C in A549 and PolII ChIA-PET in MCF7 cells. Bars indicate the average values. ns: not significant; ***P<0.0005. All p-values determined using Wilcoxon rank sum test.

(D-F) Box plots of the expression levels of all genes (left) or the highest expressed gene (right) comparing cSE-containing chromatin clusters with or without aSE/dSE and rEh-containing clusters detected by Hi-C in A549 and PolII ChIA-PET in MCF7 cells. Bars indicate the average values. ns: not significant; ***P<0.0005. All p-values determined using Wilcoxon rank sum test.

(G) Density plots of average GRO-seq signals within +/-3kb of each enhancer class (bottom) and within +/-3kb of the TSS of target genes predicted to be regulated by each or none of the enhancer classes (top) using PolII ChIA-PET in MCF7 cells.
